# MicroRNAs in Skeletal Muscle and Hints on Their Potential Role in Muscle Wasting During Cancer Cachexia

**DOI:** 10.3389/fonc.2020.607196

**Published:** 2020-11-24

**Authors:** Gioacchino P. Marceca, Giovanni Nigita, Federica Calore, Carlo M. Croce

**Affiliations:** ^1^ Department of Clinical and Experimental Medicine, University of Catania, Catania, Italy; ^2^ Department of Cancer Biology and Genetics and Comprehensive Cancer Center, The Ohio State University, Columbus, OH, United States

**Keywords:** cancer cachexia, skeletal muscle wasting, microRNAs, extracellular vesicles, long non-coding RNAs, ADAR

## Abstract

Cancer-associated cachexia is a heterogeneous, multifactorial syndrome characterized by systemic inflammation, unintentional weight loss, and profound alteration in body composition. The main feature of cancer cachexia is represented by the loss of skeletal muscle tissue, which may or may not be accompanied by significant adipose tissue wasting. Such phenotypic alteration occurs as the result of concomitant increased myofibril breakdown and reduced muscle protein synthesis, actively contributing to fatigue, worsening of quality of life, and refractoriness to chemotherapy. According to the classical view, this condition is primarily triggered by interactions between specific tumor-induced pro-inflammatory cytokines and their cognate receptors expressed on the myocyte membrane. This causes a shift in gene expression of muscle cells, eventually leading to a pronounced catabolic condition and cell death. More recent studies, however, have shown the involvement of regulatory non-coding RNAs in the outbreak of cancer cachexia. In particular, the role exerted by microRNAs is being widely addressed, and several mechanistic studies are in progress. In this review, we discuss the most recent findings concerning the role of microRNAs in triggering or exacerbating muscle wasting in cancer cachexia, while mentioning about possible roles played by long non-coding RNAs and ADAR-mediated miRNA modifications.

## Introduction

In humans, the skeletal muscle represents the most substantial fraction of fat-free body mass and is highly relevant to physiology. It constitutes ~40% of total body mass, encloses 50%–75% of all body proteins, and accounts for about 30%–50% of total protein turnover (1, 2), with precise percentages depending on variables like genetic factors, age, health status and nutrition (3–5). Notoriously, skeletal muscles function as effectors of the locomotor system, serve as storage for amino acids and carbohydrates, and have a central role in thermogenesis (1, 2). Also, skeletal muscles function as secretory organs since they physiologically express and release cytokines and other regulatory peptides, exerting important hormone-like effects (6).

The function and integrity of skeletal muscles can be severely impaired by increased concentrations of reactive oxygen species (ROS) or adverse conditions related to chronic diseases. These include cardiovascular diseases, cancer, chronic respiratory diseases, and diabetes (7–10). In particular, several cancer types can determine a condition of chronic systemic inflammation, which eventually leads toward the onset of cancer cachexia. From a clinical standpoint, cancer cachexia is a multifactorial metabolic syndrome that can develop progressively through different stages. It is primarily characterized by ongoing depletion of the skeletal muscle and uncontrolled weight loss (11). Other usual manifestations include loss of fat mass (at various degrees), lower energy intake, increased resting energy expenditure (REE), loss of appetite, fatigue, and resistance to chemotherapy (12).

In patients with cancer cachexia, survival is directly related to both total weight loss and rate of weight loss. Based on this, it was estimated that ~20%–30% of mortalities in these patients are due to such debilitating condition rather than the tumor itself (13, 14). The incidence and prevalence of cancer cachexia vary depending on the tumor type and stage. Recent statistical analysis on large cohorts of advanced tumor patients let emerge that pancreatic and liver cancers are malignancies at highest risk of developing cachexia (80-90%), followed by lung, gastro-esophageal, colorectal, and head-and-neck cancers (60%–80%) (12, 15). Differently, thyroid, breast, prostate, and skin cancers represent the groups at lowest risk (20%–30%) (15). Further confirmations about these statements come from a wide-transcriptome analysis of >4,500 tumor samples including 12 cancer types, which revealed a strong correlation between tumor-specific expression of cachexia-inducing factors and prevalence of the syndrome (16).

According to the knowledge acquired over the past three decades, we now know that the development of cancer cachexia is mostly driven by an aberrant tumor-induced inflammatory response. Here, the persistent release of pro-inflammatory cytokines along with immune-suppressive factors leads to systemic inflammation, with subsequent immunosuppression, debilitation, and metabolic dysfunctions (17–19). Among the most relevant factors, interleukin (IL)-6, IL-1α, tumor necrosis factor-alpha (TNFα), and interferon-gamma (INFγ) have long been recognized as mediators of cancer cachexia, though several other potential mediators have been identified (12, 16, 20, 21). In the context of skeletal muscle, these factors function as triggers of the ubiquitin-proteasome and autophagy–lysosomes pathways, which are the main responsible for the high proteolysis rate observed in muscles of cachectic individuals. Moreover, interactions of these factors with their cognate receptors at the myocyte membrane level cause a shift in gene expression toward enhanced thermogenesis and exacerbation of the inflammatory state (12, 16, 20, 21). Following this reasoning, several therapeutic agents have been developed in the attempt to prevent or block the triggering of pathways downstream of such mediators. However, despite their proven efficacy at the molecular level, no conclusive results have been obtained in terms of the effectiveness of the treatment, except for very few drugs (20–23). This is probably attributable to the multifactorial etiology of cancer cachexia, which may be more appropriately treated by the exploitation of balanced multimodal treatments (24, 25). Nevertheless, several discrepancies have been observed between experimental models of cancer cachexia and the corresponding human condition, and inferences derived from such models likely failed to faithfully recapitulate mechanistic insights related to human cachexia (21, 26). Thus, cancer cachexia still represents a challenging issue that needs to be more accurately defined, and remains underdiagnosed in many instances.

More recently, the role of non-coding RNAs (ncRNAs) in the physiopathology of striated muscle has received more considerable attention, as these molecules are involved in processes like regulation of gene expression, translational control, chromatin remodeling, and cell-to-cell communication. In particular, microRNAs (miRNAs) represent the most studied and best-characterized class of small regulatory ncRNAs to the present day. Several studies have focused on their involvement in skeletal muscle decay following the onset of specific myopathies, including cancer cachexia. As a result, it seems that miRNAs may represent valuable diagnostic/prognostic tools for cancer cachexia, as well as new potential targets for therapeutic intervention.

After illustrating their role in skeletal muscle physiology, in the present article, we give an overview of the involvement of microRNAs in mechanisms of muscle wasting during cancer cachexia. Moreover, we briefly discuss some hints on possible roles of long non-coding RNAs and A-to-I microRNA editing in the same context.

## MicroRNAs

In the current literature, ~4.6% of miRNAs are intragenic and excised from introns by the spliceosome, while >95% of the remaining are located into intergenic regions and transcribed starting from their own promoters. Only a small minority of miRNAs are located in protein-coding genomic regions (27, 28).

In their mature form, miRNAs are ~21-23 nucleotides in length and are mainly interspersed in non-coding regions of the human genome. However, mature miRNAs are not the final product of the transcription. Instead, they derive from a two-step cleavage process (29). After being transcribed by RNA polymerase II, primary miRNA transcripts (pri-miRNA) are cleaved into ∼60–80 nucleotides long RNAs by the microprocessor complex. This is essentially composed of the RNAase III Drosha and its cofactor DGCR8, a motif-specific binding protein. Drosha-cleaved RNA molecules are termed precursor miRNAs (pre-miRNAs). These typically maintain the hairpin structure presented by pri-miRNAs and are promptly exported to the cytoplasm, where the RNase type III Dicer further process them. Subsequently, two small single-stranded RNA molecules are produced, i.e., the mature miRNAs, originated from the 5′ (-5p form) and the 3′ (-3p form) arm of the pre-miRNA, respectively (29). One of these two miRNAs (termed guide strand) is loaded onto an Argonaute (AGO) protein to form the core unit of the miRNA-induced silencing complex (miRISC), while the other one (termed passenger strand) is degraded. This process is known as the canonical miRNA biogenesis pathway, by which most of the mature miRNAs are generated. However, some non-canonical biogenesis pathways also exist (29).

From the functional standpoint, miRNAs are important post-transcriptional regulators of gene expression exerting the role of translational repressors (30, 31). The basic requirement underlying such a modulatory role is a thermodynamically stable base pairing between a defined region of the miRNA sequence, referred to as the seed region, and one or multiple seed-complementary regions of an mRNA. By definition, the seed region includes nucleotides 2-8 at the 5’ terminus of miRNAs, while seed-complementary regions are usually assumed to be located within the 3’UTR (30, 31), although some exceptions to these rules have been reported in the literature (32–34). The vast majority of miRNA-mediated gene silencing is miRISC-dependent, occurs by non-perfect seed-mRNA base pairing, and requires the recruitment of additional cytoplasmic effectors, eventually causing translational repression and mRNA decay (30, 31).

MiRNAs are involved in a plethora of physiological functions and are essential for the regulation of gene expression during development, cell differentiation, and homeostasis maintenance (35–37). On the contrary, any dysfunction or alteration in their expression can lead to a wide range of pathological conditions, including cancer development (38). To date, it has been estimated that the human genome comprises ~1,900 annotated miRNA precursors giving rise to over 2,600 different mature miRNAs (39), which are thought to control the gene expression level of ~60% of human genes (40).

MiRNA expression is not homogeneous across human tissues, but is instead driven and modulated by several tissue-specific epigenetic mechanisms (41). In this regard, some conventional criteria have been proposed to classify miRNAs depending on differences in their tissue representativeness. For instance, in a wide-expression profiling study (42), miRNAs were classified as “tissue-specific” or “tissue-enriched”, depending on whether they were detected at ≥20-fold or <20-fold levels in the enriched tissue compared to the mean values for other tissues, respectively. More recently, the application of a Tissue Specificity Index (TSI) (43) on wide-transcriptome data has allowed the classification of miRNA genes into housekeepers, “intermediate” and tissue-specific (28, 44).

## Myogenic miRNAs: An Overview

The striated muscle expresses its tissue-specific miRNAs, conventionally termed myomiRs. To date, the group of ascertained myomiRs includes miR-1-3p, -133a-3p, -133b, -206, miR-208a-3p, -208b-3p, and -499a-5p (**Table 1**) (45–48), with miR-208a-3p being cardiac muscle-specific while miR-206 being skeletal muscle-specific. Indeed, recent deep-sequencing analysis has let emerge further putative myomiRs (28, 44), although no meaningful information is available concerning their function in skeletal muscle physiology.

**Table 1:** List of ascertained myomiRs and most recognized miRNAs with myogenic functions, and their physiological role within the skeletal muscle.

miRNA(tissue-specificity)	miRNA transcript(chromosome band)	Clustered with(chromosome band)	Host gene(biotype)	Reported function in skeletal muscle physiology
**miR-1-3p** **(myomiR)**	hsa-mir-1-1 (20q13.33) hsa-mir-1-2 (18q11.2)	hsa-mir-133a-2 (20q13.33) hsa-mir-133a-1 (18q11.2)	MIR1-1HG (mRNA) MIR133A1HG (lncRNA)	Mainly implicated in the regulation of myogenesis and cell cycle progression. Promotes differentiation of myoblast and satellite cell and prevents their proliferation by targeting PAX7, PAX3, YY1, HDAC4, Dll-1, NOTCH3, MEOX2, IGF1, POLA1, CCND1, and CCND2. Indirectly causes downregulation of TGFβ/Mstn-SMAD signaling. Regulates embryonic morphogenesis, cytoskeleton reorganization, and cell cycle progression by targeting NFAT5, MAP4K3, FZD7, and RARB. Alters chromatin structure by targeting SMARCD1 and SMARCB2. Shares most of its biological function with miR-206.
**miR-133a-3p** **(myomiR)**	hsa-mir-133a-1 (18q11.2) hsa-mir-133a-2 (20q13.33)	hsa-mir-1-2 (18q11.2) hsa-mir-1-1 (20q13.33)	MIR133A1HG (lncRNA) MIR1-1HG (mRNA)	Involved in the regulation of myoblast and satellite cell proliferation/differentiation. Partially counterbalances the biological role of miR-1/206. Promotes the proliferative state and downregulates expression of myomiRs by targeting SRF. Promotes differentiation by targeting FoxL2 and nPTB. Controls the myogenic program by targeting GLI1 and GLI3. Modulates cytoskeleton reorganization, cell growth and cell cycle progression by targeting RHOA, CDC42, DYNAMIN-2, CALCINEURIN, SP1, and IGF1R. Regulates thermogenesis and energy expenditure by targeting UCP2.
**miR-206** **(myomiR)**	hsa-mir-206 (6p12.2)	hsa-mir-133b (6p12.2)	–	Skeletal muscle-specific. Not expressed in cardiac muscle. Mainly implicated in the regulation of myogenesis and cell cycle progression. Promotes differentiation of myoblast and satellite cell and prevents their proliferation by targeting PAX7, PAX3, HDAC4, NOTCH3, MEOX2, IGF1, POLA1, CCND1, and CCND2. Indirectly causes downregulation of TGFβ/Mstn-SMAD signaling. Regulates embryonic morphogenesis, cytoskeleton reorganization, and cell cycle progression by targeting CLCN3, NFAT5, MAP4K3, FZD7, and RARB. Alters chromatin structure by targeting SNAI2, SMARCD1 and SMARCB2. Shares most of its biological function with miR-1-3p.
**miR-133b** **(myomiR)**	hsa-mir-133b (6p12.2)	hsa-mir-206 (6p12.2)	MIR133BHG Or LINCMD1 (lncRNA)	Biological function largely overlapping with that of miR133a. Controls the myogenic program by targeting GLI1. Modulates cytoskeleton reorganization, cell growth and cell cycle progression by targeting RHOA, CDC42, and SP1. Might be dispensable for development, function, and regeneration of skeletal muscle.
**miR-499a-5p** **(myomiR)**	hsa-mir-499a (20q11.22)	–	MYH7B (mRNA)	Controls the skeletal muscle energetic-oxidative status together with miR-208b and causes a switch from type II to type I myofibers by targeting SOX6, PURβ, SP3, and HP-1β. Probably implicated in modulation of satellite cell differentiation by targeting MEF2C.
**miR-208b-3p** **(myomiR)**	hsa-mir-208b (14q11.2)	–	MYH7 (mRNA)	Controls the skeletal muscle energetic-oxidative status together with miR-499a and causes a switch from type II to type I myofibers by targeting SOX6, PURβ, SP3, and HP-1β.
**miR-486-5p** **(muscle-enriched)**	hsa-mir-486-1 (8p11.21)	–	ANK1 (mRNA)	Primarily involved in myocyte and satellite cell differentiation by targeting of PAX7, PAX3, and MSTN. Promotes skeletal muscle growth and hypertrophy by targeting PTEN, FOXO1, and MSTN.
**miR-29a-3p**	hsa-mir-29a (7q32.3)	hsa-mir-29b-1 (7q32.3)	–	Primarily involved in myocyte and satellite cell differentiation. Promotes myogenic gene expression during myoblast differentiation by targeting YY1, RYBP, AKT2, and AKT3. Targeting of AKT2/3 also negatively modulates muscle growth and cell cycle progression.
**miR-29b-3p**	hsa-mir-29b-1 (7q32.3) hsa-mir-29b-2 (1q32.1)	hsa-mir-29a (7q32.3) hsa-mir-29c (1q32.1)	– MIR29B2CHG (lncRNA)	Primarily involved in myocyte and satellite cell differentiation. Promotes myogenic gene expression during myoblast differentiation by targeting YY1, RYBP, AKT2, and AKT3. Targeting of AKT2/3 also negatively modulates muscle growth and cell cycle progression.
**miR-29c-3p**	hsa-mir-29c (1q32.1)	hsa-mir-29b-2 (1q32.1)	MIR29B2CHG (lncRNA)	Primarily involved in myocyte and satellite cell differentiation. Promotes myogenic gene expression during myoblast differentiation by targeting YY1, RYBP, AKT2, and AKT3. Targeting of AKT2/3 also negatively modulates muscle growth and cell cycle progression.
**miR-23a-3p**	hsa-mir-23a (19p13.2)	hsa-mir-27a (19p13.2) hsa-mir-24-2 (19p13.2)	LOC284454 (lncRNA)	Opposes muscle atrophy by targeting MURF1 and MAFbx. Promotes muscle growth, hypertrophy and cell cycle progression by targeting MSTN, SMAD3, PTEN, and FOXO1. Might be dispensable for myocyte differentiation and skeletal muscle formation.
**miR-27a-3p**	hsa-mir-27a (19p13.2)	hsa-mir-23a (19p13.2) hsa-mir-24-2 (19p13.2)	LOC284454 (lncRNA)	Promotes muscle hypertrophy by targeting MSTN. Modulates myogenic gene expression by targeting MEF2C and PAX3. Dowregulates glycogenolysis and indirectly alters mitochondrial functionality by targeting PGM2 and GAA.
**miR-23b-3p**	hsa-mir-23b (9q22.31)	hsa-mir-27b (9q22.31) hsa-mir-24-1 (9q22.31)	AOPEP (mRNA)	Seems to exert the same biological function of miR-23a-3p. Might be dispensable for myocyte differentiation and skeletal muscle formation.
**miR-27b-3p**	hsa-mir-27b (9q22.31)	hsa-mir-23b (9q22.31) hsa-mir-24-1 (9q22.31)	AOPEP (mRNA)	Seems to exert the same biological function of miR-27a-3p. Might be dispensable for myocyte differentiation and skeletal muscle formation.
**miR-24-3p**	hsa-mir-24-2 (19p13.2) hsa-mir-24-1 (9q22.31)	hsa-mir-23a (19p13.2) hsa-mir-27a (19p13.2) hsa-mir-23b (9q22.31) hsa-mir-27b (9q22.31)	LOC284454 (lncRNA) AOPEP (mRNA)	Positively regulates myogenesis and indirectly promotes skeletal muscle repair by targeting SMAD2. Might be dispensable for myocyte differentiation and skeletal muscle formation.

MyomiR expression follows a distinct spatio-temporal pattern (49–51), aiming to properly regulate myogenesis, satellite cell differentiation, protein turnover, and muscle repair. Besides myomiRs, however, a subset of non-muscle-specific miRNAs also exert essential myogenic functions, including, for example, miRNAs of the miR-29 family (52), the miR-23a/b clusters (53), and miR-486-5p (48) (**Table 1**).

Overall, some of these miRNAs are clustered together and transcribed as bicistronic (46) or polycistronic (53) transcripts, whereas others are monocistronic and transcribed independently (47). Also, some of these miRNAs are intragenic, and their expression rate primarily depends on that of their host gene (47). In general, however, myomiR expression is mainly controlled by a set of muscle-specific transcription factors and cofactors, referred to as myogenic regulation factors (MRFs). Several MRFs are characterized by the basic helix-loop-helix (bHLH) motif and include myoblast determination protein (MyoD), myogenic factor 5 (Myf5), Myf6, and Myogenin. Others contain the MADS-box motif and include the myocyte enhancer factor-2 (Mef2) family of transcription factors (54, 55). Non-muscle-specific miRNAs with myogenic functions, instead, are often transcribed by a subset of non-myogenic transcription factors, albeit MRFs exert a significant influence on their expression.

### Transcriptional Regulation of Myogenic miRNAs

The miR-1 (miR-1/miR-206) and miR-133 (miR-133a/miR-133b) families certainly represent the best-studied group of myomiRs. These consist of three bicistronic transcripts, specifically mir-1-1/mir-133a-2, mir-1-2/mir-133a-1, and mir-206/mir-133b, clustered into three different chromosome loci of the human genome and giving rise to four distinct mature miRNAs (46). Because of high sequence similarity, miR-1 shares a large fraction of targets with miR-206 and miR-133a with miR-133b. Intriguingly, these miRNAs are capable of regulating their expression through feedback loop mechanisms in several circumstances, as described hereafter. Mir-208b and mir-499a are instead monocistronic transcripts hosted in intronic regions of genes encoding for two isoforms of myosin heavy chain-β (β-MHC), i.e., myosin heavy chain 7 (MYH7) and MYH7B, respectively (47, 56).

MyoD, Mef2, and serum response factor (SRF) are notoriously responsible for transcription of myomiRs (55, 57, 58). Meanwhile, each of these MRFs is regulated by other factors. Concerning MyoD, it was demonstrated that its stability is under the control of the mammalian target of rapamycin complex-1 (mTORC1). In contrast, the blockage of mTOR kinase activity results in its degradation, with consequent underexpression of miR-1, -206, -499a, and several non-muscle-specific miRNAs (59). MyoD is also controlled by the paired box 7 (Pax7) transcription factor through a dual mechanism consisting of downregulation of MyoD expression and impairment of its transcriptional activity (60). The latter, in particular, involves the expression of two transcriptional targets of Pax7 and Pax3, namely inhibitor of DNA binding 2 (ID2) and ID3, which antagonize myogenic bHLH transcription factors (61, 62). Pax7/3-mediated control of MyoD is fundamental to allow the proliferation of skeletal muscle precursors and to maintain the status of quiescent satellite cells (61, 62). On the other hand, however, both Pax7 and Pax3are direct targets of miR-1/206 and miR-486, and their miRNA-mediated suppression occurs during the early phase of myogenic differentiation, when the expression of these miRNAs considerably increases (62–65). Also, miR-1, -133, -206, -29b-2, 29c, and Pax7 are under the control of Yin Yang1 (YY1), a ubiquitously expressed transcription factor positively regulated by the nuclear factor-kappaB (NF-κB) signaling (52, 66). Precisely, activation of the NF-κB signaling causes increased expression of YY1, which in turn causes the upregulation of Pax7 expression and the subsequent repression of skeletal myogenesis and satellite cell differentiation. At the same time, YY1 is a direct target of miR-1 and miRNAs of the miR-29 family (miR-29a/b/c), which suppress its expression during the early differentiation phase (52, 66).

Local chromatin remodeling undoubtedly exerts an essential role in the control of MRFs-mediated miRNA gene expression in skeletal muscles. For instance, it is known that the Snail DNA-binding proteins Snai1 and Snai2 act as transcriptional repressors of MYOD by recruiting histone deacetylase 1 and 2 (HDAC1/2) onto genes containing G/C-rich E box motifs, which are almost exclusively associated with differentiation. On the contrary, Snail-HDAC1/2 complexes are not recruited in MyoD-targeted genes containing A/T-rich E box motifs. Such an occurrence causes the blockage of MyoD-induced myogenic differentiation but does not prevent MyoD function in cell growth (67). In this context, miR-206 exerts an essential role in the switch from myoblast growth to myoblast differentiation by directly targeting SNAI2, while miR-30a inhibits expression of SNAI1. Therefore, it was noticed that the absence of such a regulatory loop would impede myogenic differentiation (67).

The regulation of Mef2 represents another important mechanism capable of modulating myogenic miRNA expression. Here, HDAC4 plays an essential role as a repressor of both Mef2 activity and myogenic miRNAs expression, and represents a point of convergence of several pathways. For example, the binding of HDAC4/5 and Mef2-interacting transcriptional repressor (MITR) to Mef2 hinder Mef2 transcriptional activity (68–70). However, upon increased concentrations of intracellular calcium ions (Ca^2+^), calmodulin-dependent protein kinase (CaMK) causes the dissociation of both these inhibitors from Mef2, rehabilitating its activity. Also, CaMK phosphorylates Mef2 cooperatively with p38, a member of the mitogen-activated protein (MAP) kinases, maximizing Mef2-mediated transcription (69, 70). The alpha subunit of the heterotrimeric G protein complex (Gαi2) also suppresses HDAC4 *via* an indirect mechanism involving the protein kinase C (PKC) signaling (71).

Importantly, HDAC4 is a direct target of several miRNAs with myogenic function, such as miR-1/206 and miRNAs of the mir-29 family (49, 72, 73). This generates further regulatory loops capable of determining the switch from proliferation to differentiation of myoblasts. One of such loops involves the control of Mef2/MyoD activity by transforming growth factor-beta (TGFβ) and myostatin (Mstn), two established negative regulators of MyoDand Mef2 and suppressors of myoblast differentiation (74–76). Interaction of TGFβ or Mstn with their cognate receptor induces phosphorylation of small mother against decapentaplegic 2 (SMAD2) and SMAD3 with consequent activation of the SMAD2/3/4 complex. The latter then translocates to the nucleus, where it functions as a transcription factor that inhibits the expression of miR-206 while favoring the upregulation of HDAC4 (73). Moreover, activated SMAD3 induces the recruitment of a nuclear complex composed of YY1, Ring1-YY1-binding protein (Rybp), and Polycomb-repressive complex (PRC), capable of negatively regulating the expression of several miRNAs with myogenic functions (77, 78). Conversely, under normal myogenic differentiation conditions, increased concentrations of both miR-206 and miR-29(a/b/c) prevent the induction of SMAD3 by an indirect mechanism and cause a decrease in basal levels of SMAD3 (73), eventually leading to the downregulation of HDAC4, YY1, Rybp, and PRC (77, 78). MiR-29 also targets the RYBP 3’UTR, leading to the rapid upregulation of genes involved in somitic myogenesis and differentiation (77).

The Notch signaling represents another critical control point of myomiR expression as it prevents the activity of bHLH transcription factors and is determinant for the maintenance of quiescence in myoblasts (79–81). The binding of ligands with Notch receptors stimulates their proteolytical cleavage, causing the release of the Notch intracellular domain (ICD). The last shuttles into the nucleus, where it interacts with the recombination signal binding protein for immunoglobulin kappa J region (RBPJ). This event lastly leads to the formation of a Notch ICD-RBPJ transcriptional complex (80, 81), which antagonizes bHLH transcription factors (79). In this context, the Numb protein causes degradation of Notch1 in differentiating myoblasts and satellite cells (82, 83), allowing the bHLH-dependent transcription. In turn, Numb is negatively regulated by direct targeting of miR-146a, which thus turns the scale in favor of quiescence (84). MiR-1 can regulate the Notch signaling by inducing transcriptional repression of the Notch ligand Delta-like 1 (DLL-1) (85). Moreover, miR-1/206 modulate NOTCH3 levels during the later phase of differentiation by direct targeting of its 3’UTR, subsequently restoring Mef2 expression and p38 functionality (86, 87).

FoxO3, a non-myogenic transcription factor, was proved to be directly involved in the induction of miR-1/133a expression (88). FoxO3 is under the control of the insulin-like growth factor 1 (IGF1) – RAC serine/threonine-protein kinase Akt (Akt) signaling pathway, notoriously involved in muscle growth. The binding of IGF1 to its receptor (IGF1R) leads to activation of Akt, which in turn phosphorylates and inactivates FoxO3, causing a reduction in miR-1 expression. On the other hand, IGF1 is an established target of miR-1/206 (88, 89), and IGF1R transcript is directly targeted by miR-133a (90), giving rise to a reciprocal regulation between miR-1/206 and IGF1-Akt-FoxO3 signaling during myogenesis. MiR-29 also takes part in this regulatory feedback mechanism by directly suppressing the expression of AKT3/2 (91, 92).

Interestingly, *in vitro* experiments showed that sphingosine-1-phosphate (S1P) might cause a significant delay in the expression of miR-1, -206, and -486 by activation of sphingolipid signaling (93). However, no details were retrieved concerning the precise molecular mechanism.

### Post-Transcriptional Regulation of Myogenic miRNAs

Regulation of myogenic miRNAs can also occur at the post-transcriptional level through mechanisms modulating either their processing or availability. For example, it was demonstrated that muscleblind-like splicing regulator 1 (MBNL1), a protein involved in alternative RNA splicing, regulates the processing of miR-1 specifically at the pre-miRNA level by binding to its loop region. This protects the pre-mir-1 loop region from post-transcriptional modifications (94). In contrast, nuclear sequestration of MBNL1 in individuals with myotonic dystrophy causes the replacement of MBNL1 with Lin28. The latter promotes the uridylation of the pre-miR-1 loop region, subsequently rendering pre-miR-1 resistance to Dicer cleavage (94). An analogous situation presumably occurs following MBNL1 suppression (95).

Bone morphogenetic proteins (BMPs), members of the TGFβ superfamily, regulate proliferation, differentiation, and apoptosis of various types of cells and organs, including skeletal muscles. For instance, BMPs are capable of preventing terminal differentiation of myogenic cells by inhibiting the transcription of the muscle-specific nuclear factors MyoD and Myogenin (96), thereby impacting on myogenic miRNA transcription. However, among BMPs, BMP2 was also shown to influence miRNA processing (97). Specifically, the interaction between BMP2 and its cognate receptor stimulates phosphorylation and activation of SMAD1. Phosphorylated SMAD1 interacts with SMAD4, forming the SMAD1/4 complex. The latter shuttles into the nucleus and impedes Drosha-mediated cleavage of miR-206 through an undefined mechanism. As a result, miR-206 is accumulated into the nucleus in the form of pri-mir-206, whereas concentrations of cytosolic miR-206 significantly decrease (97).

One further post-transcriptional regulatory mechanism reported to control the expression of myomiRs depends on KH-type splicing regulatory protein (KSRP), which was specifically demonstrated to regulate the processing of the miR-1 and miR-206 families of myomiRs in C2C12 cells (98).

Intriguingly, it was demonstrated that TAR DNA-binding protein 43 (TDP-43) physically associates with mature miR-1 and miR-206 in individuals with amyotrophic lateral sclerosis. This prevents their loading onto AGO proteins and thus decreases their availability, impeding them to exert their function as gene expression silencers (99).

### Functions of Myogenic miRNAs in Skeletal Muscle Physiology

MyomiRs of the miR-1 and miR-133 families have a profound impact on skeletal muscle physiology, as they allow the fine-tuning of processes related to skeletal myogenesis and muscle regeneration (100–102). Meanwhile, several non-muscle-specific miRNAs, including miR-29 and -486, also cooperate in determining the switch between myoblast quiescence, proliferation, and differentiation (52, 62).

Besides targeting inhibitors of myogenic gene expression, microarray analysis demonstrated that miR-1 and -206 repress the expression of a small set of genes controlling muscle structure and function (103). Among the validated targets, the mesenchyme homeobox 2 (MEOX2) transcription factor has an established key role in somitogenesis (104). At the same time, chloride voltage-gated channel 3 (CLCN3) is involved in the regulation of cell volume and fibroblast-to-myofibroblast transition (105). MAP4K3, frizzled class receptor 7 (Fzd7), nuclear factor of activated T-cells 5 (NFAT5), and retinoic acid receptor beta (RARB) are non-muscle-specific components involved in embryonic morphogenesis, cytoskeletal changes, cell growth and differentiation (106–108). Smarcd1 and Smarcb2 are two well-known non-muscle-specific chromatin remodeling factors that might have a role in satellite cell differentiation and cytoskeletal reorganization (109).

MiR-1 and -206 also target a few cellular components involved in cell cycle progression. For instance, it was demonstrated that both these miRNAs directly target the *POLA1* gene (50), encoding for the alpha 1catalytic subunit DNA polymerase, as well as two members of the cyclin family, i.e., cyclin D1 (*CCND1*) and *CCND2* (110, 111), which function as positive regulators of the cyclin-dependent (CDK) kinases. MiR-206 might significantly impair the cell size in myogenic lineage through the inhibition of HDAC4 activity (112). In particular, an alteration of endogenous miR-206 expression associated with the hypertrophy and atrophy of muscles in mice. Nonetheless, manipulation of the miR-206/HDAC4 axis had no significant effect in post-natal muscle mass or adaptive responses.

MiR-133a takes part in myoblast differentiation as well, and it partially counterbalances the biological role of miR-1 (85). For instance, miR-133a directly targets the 3’UTR of SRF (49), required for skeletal muscle growth and maturation (113), thus causing its silencing and maintaining myoblasts and satellite cells in a proliferative state (49, 85). On the other hand, miR-133a targets the FOXL2 transcription factor (114) as well as the alternative splicing factor neuronal polypyrimidine tract-binding protein (nPTB) (115), promoting differentiation. Moreover, miR-133a was recently demonstrated to guide the myogenic program by exerting direct control over components of the Hedgehog pathway, including the GLI1 and GLI3 transcription factors. In contrast, miR-133a knockdown impaired myotome formation (116). Along with proliferation and differentiation, miR-133a also regulates the reorganization of the actin cytoskeleton and hypertrophy of striated muscles. This is achieved by the direct targeting of RAS homolog family member A (RHOA), Cell division cycle 42 (CDC42) (117), DYNAMIN-2 (118), and CALCINEURIN (119). Still, miR-133a is involved in the control of cell cycle – through transcriptional repression of Sp1, responsible for the expression of CCND1 (120) – thermogenesis and energy expenditure – by direct targeting of uncoupling protein 2 (UCP2), a mitochondrial anion carrier that affects myoblast differentiation (121).

Differently from miR-133a, very few pieces of evidence exist concerning the precise biological role of miR-133b in skeletal muscle physiology. However, studies from cancer research reveal that a large fraction of miR-133b targets overlaps with those of miR-133a, including FOXL2 (122), GLI1 (123), RHOA, CDC42 (124, 125), and SP1 (126), indicating overlapped biological functions. Nonetheless, knockout experiments performed on a murine model suggested that, indeed, the miR-206/133b cluster might be dispensable for development, function, and regeneration of skeletal muscle, probably because of compensation by miR-1/133a (127). Analyses performed on the soleus and plantaris muscles in mice showed that overload-induced hypertrophy resulted in decreased expression of miR-1 and miR-133a, and a parallel increase of muscle weight, hence suggesting that such miRNAs might play a regulatory role in mediating skeletal muscle response to functional overload (128).

In the context of skeletal muscles, expression of miR-208b and -499a is restricted to type I (slow-twitch) muscle fibers (47) and partly depends on the estrogen-related receptor-gamma and beta (ERRγ/β) (129). In contrast, peroxisome proliferator-activated receptor-alpha (PPARα) was shown to repress their expression (129). Accordingly, these two myomiRs determine a switch in myofiber gene program (from type II to type I) and control the skeletal muscle oxidative status by silencing the expression of a shared set of transcriptional repressors of β-MHC genes, consisting of SOX6, PURβ, SP3, and HP-1β (47, 129). In turn, activation of the type I myofiber gene program creates a positive regulatory loop *via* the expression of *MYH7* and *MYH7B*, further reinforcing the slow-twitch muscle gene program. In addition, miR-499a might be implicated in the regulation of muscle differentiation by direct targeting of MEF2 isoform C (130).

**Figure 1:**
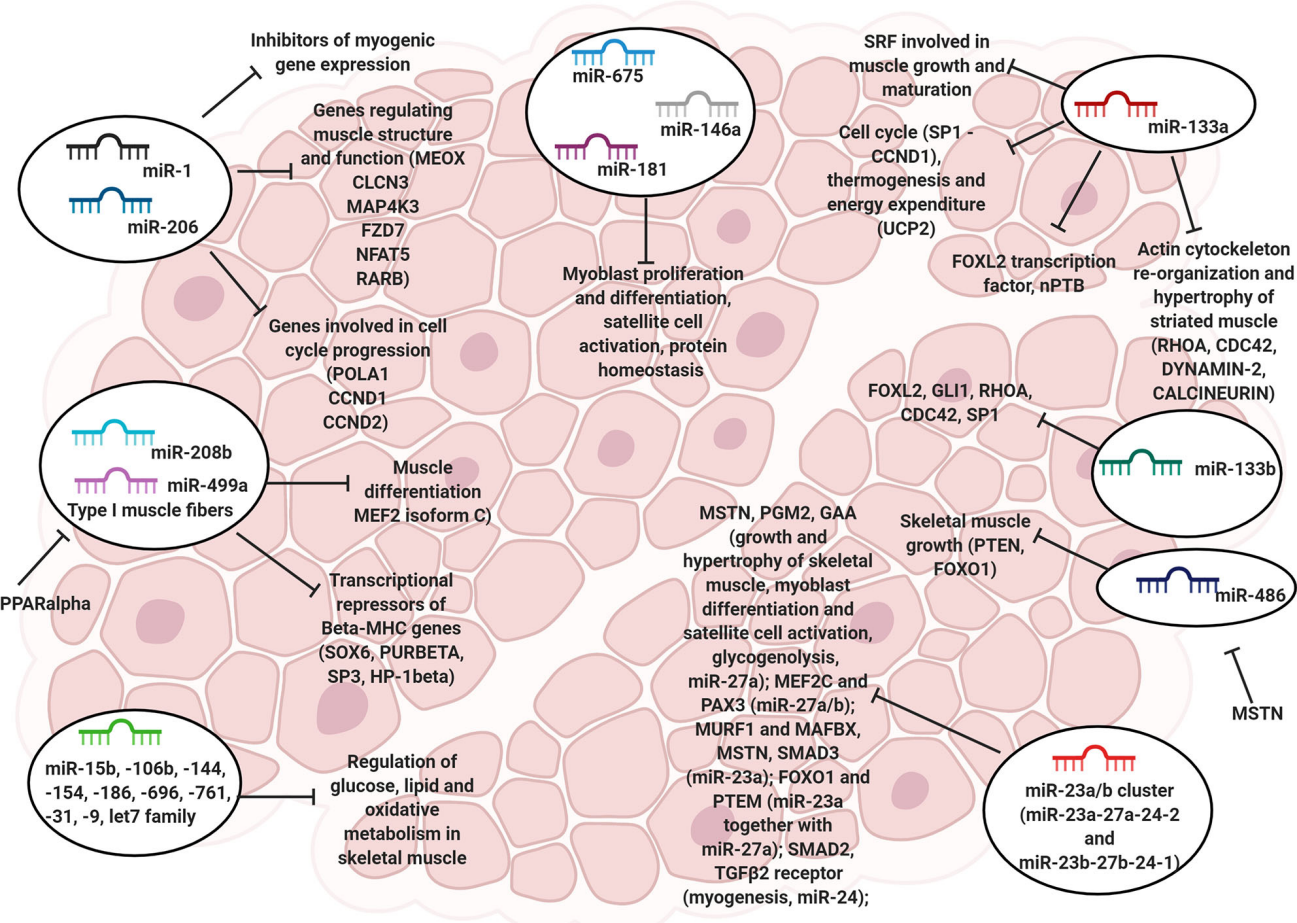
The role of myomiRs in skeletal muscle. Representative image showing the miRNAs involved in the regulation of skeletal muscle cell physiology, including proliferation, differentiation, satellite cell activation and cytoskeleton re-organization.

A miRNA profiling performed on side population cells (a cell type that plays a crucial role in muscle regeneration after injury along with satellite cells) allowed the identification of a set of overexpressed molecules compared to main population cells (131). In particular, the overexpression of miR-128 in mice led to an impairment of cell proliferation and differentiation. Further analyses revealed that miR-128a mediated the maintenance of the quiescent state and regulation of cell differentiation through the modulation of genes involved in myogenesis, adipogenesis, and osteogenesis including Pax3, Runx1, and PPAR (131). The same authors later found that miR-128a also regulated genes involved in insulin signaling (such as *Irs1*) and that its inhibition mediated by TNF-α resulted myotube maturation and myofiber hypertrophy both *in vivo* and *in vitro* (132), paving the way for further investigations in human as miR-128 is conserved among species. MiR-486 is a potent regulator of the IGF1-Akt-mTORC1 pathway (48), which plays a major role in skeletal muscle growth (133). In particular, such regulation is achieved by direct targeting of phosphatase and tensin homolog (PTEN) and FOXO1, two negative modulators of the phosphoinositide-3-kinase (PI3K)-Akt signaling (48). In turn, the expression of miR-486 is negatively controlled by Mstn, which functions as an autocrine factor capable of inhibiting myogenesis (134). Accordingly, the overexpression of miR-486 was shown to induce striated muscle hypertrophy in mice knockdown for Mstn (134), while transfection of miR-486 mimic allowed the rescue of muscle mass in atrophic skeletal muscles (120). In a murine model of chronic kidney disease, characterized by increased muscle protein degradation mediated by E3 ligases, Atrogin-1/MAFbx, and MuRF-1, ectopic expression of miR-486 resulted in impaired skeletal muscle atrophy by the blockage of FoxO1 translation (135) (**Figure 1**).

Similarly to miR-29 and -486, the miR-23a/b clusters (miR-23a–27a–24-2 and miR-23b–27b–24-1) are also enriched in skeletal muscle (53, 136), exerting important regulatory roles. In particular, miR-27a assists both growth and hypertrophy of skeletal muscle by silencing MSTN expression and subsequently promoting myoblast differentiation and satellite cell activation (137, 138). Also, miR-27a targets phosphoglucomutase (PGM2) and acid α-glucosidase (GAA), two enzymes involved in glycogenolysis, and its combined action with miR-142 regulates glycogen and fatty acid metabolism, and alters mitochondrial functionality in both myoblasts and myofibers (139). Furthermore, miR-27a/b influences the myogenic gene expression program by targeting the 3’UTR of MEF2C (140) and PAX3 (141). MiR-23a attenuates muscle atrophy by directly targeting both muscle RING finger containing protein 1 (MURF1) and muscle atrophy F box protein (MAFBX) (136, 142), two E3 ubiquitin ligases of the ubiquitin-proteasome pathway that take part in the breakdown of short-lived and regulatory proteins (143). Moreover, miR-23a suppresses expression of both MSTN and SMAD3 and acts synergistically with miR-27a to downregulate FOXO1 and PTEN and upregulate PI3K-Akt signaling, opposing the loss of muscle mass and contractile function (144). MiR-24 has a role in myogenesis (145), which is partly exerted by direct targeting of SMAD2 (146). In the context of skeletal muscle injuries, miR-24 was shown to act concomitantly with miR-122, which targets the receptor II of TGFβ. Such an event would inhibit the TGFβ-SMAD signaling, thus favoring satellite cell activation and skeletal muscle repair (135, 146) (**Figure 1**). Nonetheless, loss-of-function experiments suggested that the miR-23a/b clusters would indeed be dispensable for myogenesis and skeletal muscle function, as mice knockout for these two clusters reported only subtle effects on skeletal muscle development and adaptation after exercise endurance (147).

Besides the aforementioned myogenic miRNAs, several other miRNAs are also known to exert either essential or dispensable roles in myoblast proliferation/differentiation, satellite cell activation, and protein homeostasis, including miR-675, miR-146a, and miR-181, respectively (148). Also, an extensive list of miRNAs involved in the control of glucose, lipids, and oxidative metabolism in skeletal muscle has been reported, including miR-15b, -106b, 144, -154, -186, -696, -761, -31, -9, and miRNAs of the let-7 family (135, 139, 149) (**Figure 1**).

## Alteration of miRNA Expression in Skeletal Muscles During Cancer Cachexia

It is evident that, overall, miRNAs regulate almost all aspects of skeletal muscle physiology. Thus, any alteration in their expression or functionality is likely to result in impairments of relevant features, such as metabolic state, proteostasis, regenerative capabilities, and performance. This suggests the possibility for miRNAs to play a role as mediators and biomarkers of skeletal muscle loss in several debilitating diseases, including cancer cachexia. Accordingly, small RNA-seq analyses have been recently performed on skeletal muscle biopsies from cachectic tumor patients and mice, revealing a real correlation between changes in myogenic miRNA expression and cancer cachexia occurrence.MiRNA profiling on muscle biopsies from cachectic tumor patients

As yet, only two studies have been focused on the shift in myogenic miRNA expression in the context of human cancer cachexia (**Table 2**). In the former one, the myogenic miRNA expression profile from cachectic patients with pancreatic and colorectal cancers was compared with that from non-cachectic patients suffering from the same tumor types (150). The authors identified and validated eight differentially expressed miRNAs, i.e., let-7d-3p, miR-199a-3p, -1296-5p, -345-5p, -3184-3p, -423-5p, -423-3p, and -532-5p, between the two conditions. Precisely, these eight miRNAs were significantly upregulated in the muscles of cachectic patients and showed both predictive and prognostic value. The authors then identified 147 potential target genes in the cachectic condition, mainly related to myogenesis, inflammation, innate immune response, and signaling pathways involved in morphogenesis and development (150).

**Table 2:** List of significantly deregulated miRNAs in skeletal muscles of cachectic individuals suffering from tumors.

Organism (Study)	Tumor type	miRNAs	Alteration type	Known miRNA functions in the skeletal muscle
Human (Narasimhan et al. (150))	Pancreatic and colon cancer	let-7d-3p, miR-199a-3p, miR-345-5p, miR-423-5p, miR-423-3p, and miR-532-5p, miR-1296-5p, miR-3184-3p	Upregulated	None
Human (Van de Worp et al. (151))	Non-small cell lung cancer	miR-335-3p, miR-424-5p, miR-424-3p, miR-450a-5p, miR-450b-5p	Upregulated	None
		miR-15b-5p, miR-20a-3p, miR-26a-2-3p, miR-103-3p, miR-144-5p, miR-370-3p, miR-379-5p, mir-409-3p, miR-451a, miR-483-5p, miR-483-3p, miR-485-3p, miR-512-3p, miR-517a-3p, miR-517c-3p, miR-518b, miR-519a-3p, miR-520g-3p, miR-520h, miR-522-3p, miR-656-3p, miR-766-3p, miR-1255b	Downregulated	miR-15b-5p negatively modulates myogenesis and cell proliferation; promotes muscle stem cell quiescence; modulates mitochondrial-dependent apoptosis; downregulates the insulin-PI3K-AKT signaling. miR-451a inhibits myogenic differentiation; regulates lipid metabolism; regulates energetic state and mitochondrial activity.
Mouse (Soraes et al. (152))	C26 colon carcinoma	miR-133a-3p, miR-140, miR-489, miR-519e, miR-7029	Upregulated	miR-133a-3p modulates myogenesis; regulates cytoskeletal organization.
		Let-7f-5p, let-7g-5p, let-7i-5p, miR-1-3p, miR-15a-5p, miR-15b-5p, miR-23a-3p, miR-23b-3p, miR-24-3p, miR-26b-5p, miR-27a-3p, miR-27b-3p, miR-143-3p, miR-195a-5p, miR-199a-3p, miR-422b-3p, miR-497a-5p	Downregulated	miR-1-3p promotes myogenesis and prevents proliferation; regulates cytoskeletal organization and chromatin structure. miR-15a/b-5p negatively modulate myogenesis and cell proliferation; promote muscle stem cell quiescence; modulate mitochondrial-dependent apoptosis; downregulate the insulin-PI3K-Akt signaling. miRNAs of the miR-23a/b clusters prevent muscle atrophy; promote muscle hypertrophy; modulate myogenic gene expression; modulate mitochondrial acticity. miR-143-3p downregulates glycolysis.
Mouse (Lee et al. (153))	Lewis lung cancer	miR-147-3p, miR-205-5p, miR-223-3p, miR-511-3p	Upregulated	None
		miR-229a-3p, miR-431-5p, miR-665-3p, miR-1933-3p, miR-3473d	Downregulated	None
Mouse (Fernandez et al. (154))	Lewis lung cancer	miR-144-5p, miR-144-3p, miR-181c-3p; miR-379-3p, miR-451a	Upregulated	miR-144-3p reduces glucose uptake and glycolysis; modulates the insulin-PI3K-Akt signaling; indirectly influences the mitochondrial activity. miR-181c-3p promotes myoblast differentiation. miR-451a inhibits myogenic differentiation; regulates lipid metabolism; regulates energetic state and mitochondrial activity.
		miR-10b-5p, miR-29b-3p, miR-146a-5p, miR-146b-5p, miR-183-5p, miR-223-3p, miR-338-5p, miR-350-3p, miR-382-5p, miR-671-3p, 1249-3p, miR-1843a-3p, miR-3535	Downregulated	miR-29b-3p promotes myoblast differentiation and prevents cell cycle progression.

In the second study, the myogenic miRNA expression profile from cachectic patients with non-small cell lung cancer (NSCLC) was compared with that from matched healthy controls (151). Here, a signature of 28 differentially expressed miRNAs was identified, with 23 being down and five upregulated in cachectic patients. However, none of these overlapped with miRNAs identified in the earlier study, nor revealed any predictive or prognostic potential. Finally, 114 potential target genes functionally expressed in the skeletal muscle were identified, most of which were related either to muscle-specific degenerative or regenerative processes (151).

### Results from Murine Models of Cancer Cachexia

A more extensive body of evidence has been reported in the case of murine models of cancer cachexia (**Table 2**). In a preliminary work, four distinct atrophic mice models – fasting, denervation, diabetes, and C26 colon carcinoma-induced cancer cachexia – were employed to frame the role of miRNAs in skeletal muscle loss (152). Noteworthy, the authors found that the miRNA signature was peculiar to each atrophic condition. Compared to the controls, muscle samples from cachectic mice presented 22 differentially expressed miRNAs, of which 17 were down and five upregulated. Of note, miRNAs of the miR-23a/b clusters, primarily involved in the prevention of protein catabolism, were among the most downregulated. MiR-143-3p, -199a-5p, -26b-5p, as well as miRNAs of the let-7 and miR-15a/b families, were also downregulated. These are involved in the modulation of insulin cascade, PI3K-Akt signaling, and glucose metabolism (149). Finally, miR-1-3p was significantly downregulated, whereas miR-133a-3p was upregulated (152).

The myogenic miRNA profiling of skeletal muscles from cachectic mice bearing Lewis lung carcinoma (LLC) revealed nine differentially expressed miRNAs compared to controls. Among these, miR-229a-3p, -431-5p, -665-3p, -1933-3p, and -3473d were downregulated, whereas miR-147-3p, -205-5p, -223-3p, and -511-3p were upregulated (153). The subsequent pathway analysis revealed that these miRNAs are likely to be involved in processes related to cell-to-cell communication, development and morphogenesis, cell cycle, and inflammatory disease, among others (153).

More recently, a myogenic miRNA profiling from skeletal muscles of cachectic LLC-bearing mice identified 18 differentially expressed miRNAs compared to the controls, with 13 being down and five upregulated (154). In particular, miR-144-3p and -451a, involved in the modulation of mitochondrial activity, energetic state, and glucose and lipid metabolism (149), were upregulated. MiR-181c, involved in myoblast differentiation (155), was upregulated as well. In contrast, miR-29b-3p was downregulated. By applying an integrated genome-wide approach combining miRNA-mRNA sequencing data, the authors identified 131 putative target genes, mostly involved in the extracellular matrix organization, cell migration, ion transport, and FoxO signaling (154).

### Molecular Mechanisms Underlying Shifts in Myogenic miRNA Expression During Cancer Cachexia

As discussed above, dysregulation in myogenic miRNA expression usually represents the consequence of altered transcriptional mechanisms. In the context of cancer cachexia, some experimental studies suggest that the deregulation in MRFs expression or function is often the leading cause of altered miRNA expression in skeletal muscles. This would then contribute to maintain or even worsen the course of such metabolic syndrome.

One example regards tumor necrosis factor-alpha (TNFα) and TNF-weak inducer of apoptosis (TWEAK), two pro-inflammatory cytokines known to induce skeletal muscle wasting under conditions of experimental cachexia through indirect destabilization and suppression of MyoD (156, 157). This is achieved by the activation of MAPK/ERK/p38 and NF-κB signaling. p38 kinase induces activation of the CCAAT/enhancer-binding protein-beta (C/EBPβ) transcription factor, which is responsible for transcription of MAFBX and other genes involved in the ubiquitin-protease pathway (158). Activation of the NF-κB signaling, instead, leads to the overexpression of Pax7, which subsequently causes repression of MyoDand Myogenin transcriptional activity (159), as previously discussed. As a result, TNF exposition induces protein catabolism and simultaneous alteration of expression of several myogenic miRNAs, including miR-1/206, miR-451a, and -146a-5p (160, 161). The latter two miRNAs were shown to be involved in proteolytic degradation, inflammatory response, and extracellular matrix remodeling (160), while others, such as miR-361, -486, and -miR-98, were suggested to exert a role in myoblast fusion capacity (161). Similar mechanisms are assumed to occur following interaction between several other pro-inflammatory cytokines, such as interferons (INFs) and interleukins (ILs), and their cognate receptors on the myocyte membrane surface (162). Nonetheless, no concrete outcomes have been reached in this regard.

Expression of MyoD might eventually be inhibited by indirect action of heme oxygenase-1 (HO-1) following FoxO1-mediated *HO-1* expression under conditions of muscle atrophy (163). Here, carbon monoxide derived from HO-1 enzymatic activity decreases nuclear translocation of C/EBP-gamma (C/EBPδ) and prevents its binding to promoters of myogenic genes, including *MyoD* (164). Moreover, expression of HO-1 is known to cause downregulation of DGCR8 and Lin28 (29), negatively impacting the pri-miRNA processing (164).

However, although these few studies focused their attention on MyoD transcriptional regulation, none of them detected any significant alteration in the expression of myomiRs in the context of cancer cachexia. Thus, it is more likely that such deregulation in miRNA expression involves ubiquitously expressed transcriptional factors.

### Discrepancies Emerging From the Data Comparison

Despite the research efforts undertaken to elucidate the role of miRNAs in skeletal muscle loss during cancer cachexia, no conclusive results have been described yet. Downstream analyses of miRNA expression have indicated that deregulated miRNAs in cachectic muscles are usually involved in processes like cell cycle, myogenesis, inflammation, extracellular matrix organization, and myoblast fusion. However, very scarce or no overlap exists between the sets of differentially expressed miRNAs detected by the various authors. Such a low consensus might depend on multiple factors. For instance, different cancer types likely alter different molecular mechanisms, subsequently determining distinct molecular signatures and energetic states within the skeletal muscle (165, 166). In turn, miRNAs might be differentially expressed through feedback loop mechanisms in order to properly modulate fluctuations in myogenic gene expression and confer robustness in signaling outcomes for specific regulatory networks (167). Analytical bioinformatic pipelines and sampling times are also assumed to be highly influential in the determination of mRNA/miRNA signatures (154, 168).

Besides the mentioned explanations, the scarceness of experimental models capable to accurately recapitulate the molecular features of human cancer cachexia also represents a major limitation in this field of oncology (21, 26). Indeed, some studies have recently evidenced the relative inconsistency of the “traditional” xenograft models of cancer cachexia, as the molecular signatures found at the tissue level revealed sharply different patterns of gene expression compared to that obtained for human patients (169, 170). Thus, more reliable models should be considered for future studies. These include tamoxifen-induced genetically engineered mouse (GEM) (169), orthotopic models of patient-derived cancer cells (171), and syngeneic mouse models of GEM-derived tumors (172), which are currently being adopted as valuable and innovative alternatives (21).

## Role of Tumor-Derived Circulating miRNAs in SM Wasting During Cancer Cachexia

Over the last decade, several studies have pointed out that cancer-secreted extracellular vesicles (EVs) profoundly contribute to the identification of new prognostic and diagnostic factors in cancer cachexia or even to the onset and progression of such syndrome. EVs are secreted by all cell types and consist of membrane-coated particles with different size: while the acronym “EVs” refers to a very heterogeneous group of secreted vesicles in general, such particles are classified as “exosomes” when their diameter ranges from 30 to 100 nm, “microvesicles” (MVs) when their size ranges from 50 to 1,000 nm, “oncosomes” (1 to 10 μm) and “apoptotic bodies” (100 nm to 5 μm) (173, 174). EVs harbor a wide array of different molecules, which most likely reflect the physiological status of the releasing cells: lipids, proteins, DNA fragments, and RNA molecules, including non-coding RNAs such as miRNAs, long non-coding RNAs (lncRNAs), rRNAs, and tRNAs (174). EVs can be isolated from different body fluids such as plasma, serum, milk, urine, saliva, spinal fluid (175). The percentage of miRNA content within EVs sharply differs from that of cytoplasmic miRNAs in donor cells, suggesting the existence of a controlled sorting mechanism by which miRNAs are selectively recruited and packaged into vesicles (176).

Several studies have demonstrated that miRNAs can also be found in body fluids not associated to EVs: in this scenario, miRNA molecules are associated to RNA-binding proteins, such as AGO2 (the effector member of the RISC complex), which protect them from degradation and maintain their stability in the extracellular environment. The mechanisms that mediate miRNA secretion through EVs rather than a vesicle-free system are still unknown, and this could occur through a highly regulated miRNA selection (177).

Although EVs were initially considered a system used by cells to get rid of unwanted molecules, several studies have instead demonstrated that secreted particles are a useful tool for the definition of specific non-invasive cancer-associated biomarkers. Examples are exosomal miR-30d-5p and let-7d-3p from plasma, which were found to be valuable diagnostic biomarkers for non-invasive screening of cervical cancer (178); upregulated miR-1290 in the serum of patients with high grade serous ovarian carcinoma allowed to discriminate these patients from those with other malignancy types (179); miR-25-3p and miR-92a-3p were identified as potential biomarkers for liposarcoma (180).

Besides their potential prognostic or diagnostic role, secreted miRNAs have a considerable impact on mediating cell-to-cell communication as they are biologically active molecules. In fact, once secreted by a donor cell, the circulating miRNAs can be adsorbed by recipient cells, where they efficiently target the 3’UTRs of mRNAs hence modulating the cell’s response to such stimulus. For example, it was demonstrated that the exosomal transfer of miRNAs in hepatocellular carcinoma (HCC) modulates carcinogenesis and promotes transformed cell growth (181). Here, the authors identified 11 secreted miRNAs, while TAK1, a member of the mitogen-activated protein kinase kinase kinase (MAP3K) family involved in homeostasis and tumorigenesis in HCC, was selected as a potential target. When Hep3B cells were incubated with Hep3B-derived exosomes, not only TAK1 protein expression was impaired in recipient cells, but also the activation of its associated pathway JNK1 as well as cell viability. Similarly, it showed that IL-4-activated macrophages secreted miRNAs through exosomes that targeted breast cancer cells (182). Among them, miR-223 promoted the invasion of recipient cells. In another study (183), it was demonstrated the endogenous transfer of exosomes between dendritic cells, whose cargo mirrored the level of cell maturation. In 2012 (184), our group demonstrated that miR-21 and miR-29a, secreted by lung cancer cells through EVs, were transferred to surrounding macrophages at the microenvironment level: here, miRNAs promoted tumor growth and spread by binding the TLR7/8 receptor and hence activating the NF-κB pathway. This study revealed, for the first time, a different mechanism used by secreted miRNAs than the post-transcriptional repression to promptly amplify a pro-tumoral response.

**Figure 2:**
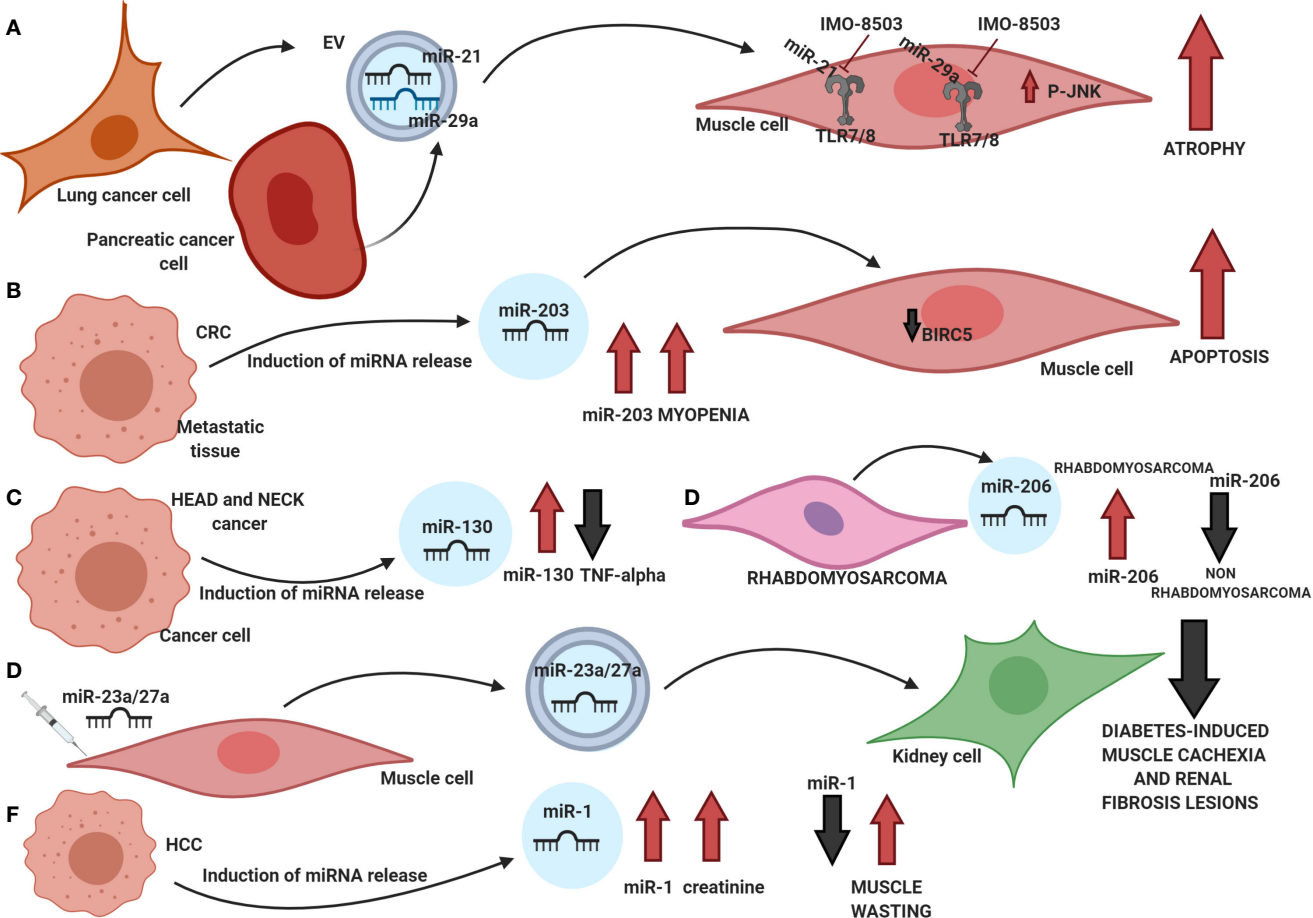
Involvement of circulating miRNAs in muscle wasting. **(A)** miR-21 and miR-29a are secreted through extracellular vesicles (EVs) by lung and pancreatic cancer cells: once internalized by myoblasts, both miRNAs bind the TLR7/8 receptor hence inducing the phosphorylation of JNK and triggering apoptosis. Such process can be inhibited by TLR7/8 inhibitor IMO-8503. **(B)** High levels of circulating miR-203 in colorectal cancer patients promote myopenia and induce apoptosis in muscle cells through the downregulation of BIRC5. **(C)** Circulating miR-130a concentration negatively correlates to TNF-α levels in the serum of head and neck cancer patients. **(D)** Circulating miR-206 can significantly discriminate between rhabdomyosarcoma and non-rhabdomyosarcoma patients. **(E)** miR-23a/27a mediate the cross-talk between muscle and kidney cells and impair both diabetes-related muscle atrophy and renal fibrosis lesions. **(F)** In hepatocellular carcinoma (HCC) patients, circulating high levels of miR-1 positively correlate with creatinine levels in the serum, while a low concentration of miR-1 is an indicator of muscle wasting.

These findings opened the doors to new investigations aimed at understanding whether cancer-secreted miRNAs modulated lean mass wasting associated with cachexia (**Figure 2**). In 2014, we demonstrated that lung and pancreatic-secreted miR-21 and miR-29a promoted atrophy in recipient murine myoblasts through their binding to the TLR7/8 receptor and the activation of the JNK pathway (185). Such a process could be significantly inhibited by IMO-8503, a TLR7, 8 and 9 antagonist (186) (**Figure 2A**). Okugawa et al. (187) showed that an elevated level of circulating miR-203 in the serum of patients who had colorectal cancer was a risk factor for myopenia and that such miRNA promoted apoptosis and impaired cell proliferation by downregulating BIRC5 (**Figure 2B**). Conversely, in head and neck cancer patients, miR-130a levels in plasma negatively correlated with TNF-α concentration (188) and allowed to discriminate between cancer patients suffering from cachexia from patients mildly malnourished with high specificity, hence displaying potential clinical utility in the diagnosis of cachexia (**Figure 2C**). In rhabdomyosarcoma, several circulating miRNAs have been identified within patient sera: miR-1, miR-133a, miR-133b, and miR-206: in particular, miR-206 was able to discriminate between rhabdomyosarcoma and non-rhabdomyosarcoma tumors with a sensitivity of 1.0 and specificity of 0.913 (189, 190) (**Figure 2D**). Zhang et al. (144) reported that miR-23a and miR-27a, two miRNAs that regulated proteins involved in the atrophy process hence reducing muscle wasting, were involved in muscle-kidney cross talk as their expression levels were increased in both serum exosomes and kidneys. Their findings suggested that high levels of miR-23a and -27a could impair diabetes-induced loss of muscle mass and reduce renal fibrosis lesions (**Figure 2E**).

On the other hand, Köberle et al. (191) evaluated the potential of serum miR-1 and miR-122 as prognostic biomarkers in patients suffering from hepatocellular carcinoma (HCC): by comparing the analyses performed on 195 sera of HCC patients and 54 patients with liver cirrhosis, the authors concluded that miR-1 significantly correlated with serum creatinine. Moreover, since miR-1 is known to be a regulator in muscle cells where it is also highly expressed, low levels of such miRNA in serum could be associated with muscle wasting and hence correlate with overall survival in advanced cancer (**Figure 2F**).

Taken together, these findings have shed light on the importance of tumor microenvironment and circulating miRNAs as relevant mediators of muscle wasting in cancer-associated cachexia.

## LncRNAs and ADAR Enzymes as Possible Players in Skeletal Muscle Loss During Cancer Cachexia

Besides the “canonical” regulatory pathways described above, further epigenetic mechanisms are emerging that allow the fine-tuning of miRNA expression, availability, and function within the skeletal muscle. Long non-coding RNAs (lncRNAs) and adenosine deaminases acting on RNA (ADAR) enzymes might represent part of such mechanisms. Interestingly, some cues from the literature suggest their active involvement in skeletal muscle atrophy. However, no clear proves exist at present concerning their involvement in the context of cancer cachexia-induced muscle atrophy.

### lncRNAs in the Skeletal Muscle

LncRNAs are 5’-capped transcripts, longer than 200 nucleotides, often spliced and polyadenylated at the 3’ terminus. LncRNAs are known to be involved in a wide range of cellular processes, such as chromatin remodeling, DNA repair, recruitment of transcriptional complexes, and enzyme activity (192). Also, lncRNAs seem to act as competitive endogenous RNAs (ceRNAs), or “sponge” RNAs, by competing with other transcripts for miRNA binding. This poses lncRNAs as relevant modulators of miRNAs availability (193).

Emerging studies have demonstrated that lncRNAs actively participate in the regulation of myogenic gene expression during skeletal myogenesis, myofiber regeneration, and muscle hypertrophy (194, 195). For instance, linc-RAM and SRA were demonstrated to foster myoblast differentiation by enhancing the formation of a transcriptional complex containing MyoD (MyoD-Baf60c-Brg1) (196) and the assembly of MyoD co-regulators (197), respectively. Linc-YY1 was found to promote myoblast differentiation and muscle regeneration by inducing the eviction of the YY1/PRC complex from the target loci and the inhibition of YY1 activity independently from PRC (198). Malat1 acts as a repressor of skeletal myogenesis and muscle repair by recruiting the Suv39h1 histone methyltransferase to MyoD-binding promoters (199). However, upon myocyte differentiation, miR-181a targets Malat1 and causes its degradation, with subsequent destabilization of the Suv39h1-repressive complex (199). Malat1 was also shown to act as a ceRNA by sponging miR-133a (200). In turn, this led toward increased expression of SRF and Mef2C, with consequent promotion of differentiation. Similar effects were reported for the miR-133b hosting transcript linc-MD1 (201). Lnc-mg sponges miR-125b, an inhibitor of IGF2 expression, whereas Gtl2 sponges miR‐135b-5p, an inhibitor of Mef2C. Thus, both these lncRNAs positively regulate muscle differentiation and hypertrophy (202, 203).

In line with these findings, several recent studies have tried to define possible correlations between lncRNA alterations and muscular diseases by examining the expression profile of lncRNAs in various relevant myopathies, including dystrophies and atrophies (195, 204). However, only one study has yet reported a correlation between the expression level of some lncRNAs and cancer cachexia-induced muscle atrophy (204). Specifically, Gtl2 and IG-DMR, both involved in genomic imprinting and muscle development, were significantly decreased in skeletal muscles of cachectic C26-bearing mice. In contrast, none of the other examined long non-coding transcripts, like linc-MD1, linc-YY1, Malat1, or SRA, showed significant deregulations compared to the other conditions (204).

Lastly, it has been demonstrated that some lncRNAs are capable of stimulating the release of tumor-derived exosomes (205). This might represent an additional critical issue in cancer cachexia. For instance, the highly upregulated in liver cancer (HULC) lncRNA, a promoter of hepatocellular carcinoma progression, was recently shown to enhance the secretion of exosomes by human hepatoma cells (205). Such a mechanism was due to the ceRNA activity of HULC, which sponged miR-372-3p. In turn, miR-372-3p is an established suppressor of Rab11a, which is a key element in exosome formation.

### ADAR1 and A-to-I RNA Editing in the Skeletal Muscle

ADAR1 and ADAR2 are conserved enzymes capable of converting adenosine (A) to inosine (I) by hydrolytic deamination. Their enzymatic function depends on their ability to interact with double-stranded RNA molecules (dsRNAs) through their dsRNA-binding domain (dsRBD) (206).

ADARs are involved in various biological processes, such as neuronal ion channel activity, increase of transcript diversity, immune response regulation, alternative splicing, RNA interference pathway, and miRNA biogenesis (206–208). The last, in particular, can be modulated by ADARs *via* editing-dependent or -independent mechanisms. In editing-dependent mechanisms, miRNA transcripts are co- or post-transcriptionally modified in their primary sequence at editing sites that prevent, or even enhance, the Drosha/Dicer-mediated cleavage (206). In editing-independent mechanisms, ADARs prevent miRNA processing by binding to their primary transcript *via* the dsRBD (209). Indeed, ADAR1 was also found to form a cytoplasmic complex with Dicer promoting pre-miRNA maturation (210). Similar findings were reported in the case of Drosha-mediated processing (211). Despite miRNA biogenesis, ADARs can also alter the function of mature miRNAs by editing their primary sequence, with particular regard to the seed region. Such an occurrence causes a change in the base-pairing ability of edited miRNAs, leading to a shift in their target repertoire (206, 212, 213).

ADARs have been demonstrated to exert essential roles for proper development. In fact, the deletion of ADAR genes caused embryonic lethality due to impaired organogenesis (*ADAR1* KO), or post-natal death (*ADAR2* KO) due to neuronal cell death (206). ADARs are not homogeneously expressed across tissues. Instead, they are subjected to spatio-temporal patterns of expression (214). In general, however, ADAR1 is ubiquitously expressed, while ADAR2 is highly expressed in the central nervous system, with much lower or null expression levels in other tissues (206).

A wide-transcriptome analysis revealed that ADAR1 is the sole responsible for RNA editing in the context of skeletal muscle. However, the A-to-I editing level in this tissue is significantly lower than in other tissues (214). ADAR1 was shown to be a regulatory constituent of myogenesis, and presumably of muscle repair. Specifically, ADAR1 contributes to the early phase of myogenesis by suppressing apoptotic processes in myoblasts, apparently in an independent manner from its catalytic activity (215). Indeed, more recent findings indicate that such a protective function might also involve the enzymatic action of ADAR1 (216, 217). In certain instances, RNA editing impedes melanoma differentiation-associated protein 5 (MDA5), a cytosolic sensor of viral RNA infection, from sensing endogenous dsRNAs as no-self, preventing the degradation of endogenous mRNAs (216, 218). Here, the p150 isoform of ADAR1 seems to exert an essential role, differently from the p110 isoform. However, both isoforms contribute to development (215, 216).

ADAR1’s editing activity is also indispensable for the regulation of expression of various genes associated with the myoblasts-myotubes transition and motility, hence influencing muscle development. This includes dynamin 1/2 (Dnm1/2) and annexin A4 (Anxa4), whose mRNAs are hyper-edited by nuclear ADAR1 and subsequently retained into the nucleus (215). A-to-I editing of the 3’UTR of Rho GTPase activating protein 26 (ARHGAP26) was instead shown to disrupt the binding sites for miR-30b-3p and miR-573, leading to increased levels of this protein (219). Moreover, a correlation analysis showed that ADAR1 also promotes MyoD and Myogenin expression, although the precise mechanism remained elusive (214).

Aminoacyl tRNA synthase complex-interacting multifunctional protein 2 (AIMP2) was identified as an important negative regulator of ADAR1 function and expression (214). AIMP2 presented significantly higher expression levels in the adult skeletal muscle compared with all other tissues, actively contributing to the low editing and expression levels of muscular ADAR1 (214). Interestingly, myomiR-1/206 were both demonstrated to base-pair with seed-complementary regions located in the 3’UTR of ADAR1, driving the temporal expression rate of ADAR1 across the skeletal myogenesis phases (215).

Some reports exist in the literature demonstrating that ADAR1 can moderately or highly edit several non-muscle specific miRNAs in skeletal muscles under physiological conditions (220–222). On the contrary, very low (<5%) (222) or no A-to-I editing was reported for myomiRs. However, the actual implications for miRNA biogenesis and targeting of such A-to-I editing events remain unknown, and no data have been reported about potential changes in miRNA editing levels during muscle atrophy.

Although no study has yet investigated the role of ADAR1 in skeletal muscles during cancer cachexia, one study demonstrated that ADAR1 is increasingly expressed and activated in skeletal muscles exposed to inflammatory stressors (223). These include TNFα, IFNγ, and TLR4, which are notorious mediators of muscle atrophy under conditions of experimental cancer cachexia (21). This event might depend on the significant decrease in myomiR expression detected under the same condition. Meanwhile, MyoD and Myogenin expression, as well as levels of active phosphorylated Akt, were shown to correlate positively with ADAR1 expression (223), in agreement with data reported by Tan et al. (214). Collectively, these data suggest a role for ADAR1 as a buffer of inflammatory stress in the skeletal muscle, by limiting muscle atrophy and promoting protein synthesis.

## Conclusions

Despite the progress concerning the mechanisms underlying experimental cancer cachexia, this syndrome still represents a significant problem for the treatment of many tumor patients and remains mostly underdiagnosed at the clinical level. Unfortunately, the actual relevance of most of the conventional mediators of cancer cachexia has remained undefined or even controversial, impeding the establishment of effective therapeutic options. For such reason, cancer investigations are trying to identify novel mediators of cancer cachexia that are exploitable as both biomarkers of disease and targets of innovative therapies.

In recent times, miRNAs have emerged as essential regulators in skeletal muscle development and homeostasis. Accordingly, alterations in their expression rates were demonstrated to decrease muscle repair abilities and worsen muscle atrophy. In this context, several cues indicate that both myogenic and tumor-derived miRNAs might play a fundamental role in muscle wasting during cancer cachexia, hence representing potential biomarkers with predictive and prognostic value, as well as novel therapeutic targets. However, the findings are still preliminary and mainly based on experimental models of cancer cachexia.

MiRNA are, in turn, finely regulated by several epigenetic components, whose dysregulation alters their expression and function. One of such components is represented by lncRNAs, which directly influence the transcriptional activity of essential myogenic transcription factors like MyoD, Myogenin, and sponge myogenic miRNAs through their seed-complementary sequences. ADAR1 represents another essential epigenetic element, capable of modulating miRNA biogenesis and function either *via* editing-dependent or -independent mechanisms. However, albeit these mechanisms have been investigated in the skeletal muscle under physiological conditions, only a few cues exist concerning their influence on miRNAs during skeletal muscle atrophy.

Overall, these facts highlight the need to establish the real role of miRNAs in skeletal muscle cell death during cancer cachexia. This might lead to the identification of new reliable biomarkers of skeletal muscle wasting in cancer patients. Moreover, the precise role of miRNA modulators should also be studied to gain a better view of the complex network governing the myogenic gene expression throughout this debilitating syndrome.

## Author Contributions

GM, GN, and FC wrote the manuscript. GM, GN, and FC contributed to the editing of the manuscript as needed while CC critically revised our work. All authors contributed to the article and approved the submitted version.

## Acknowledgments

Figures of this manuscript were realized by using the software BioRender.

## Conflict of Interest

The authors declare that the research was conducted in the absence of any commercial or financial relationships that could be construed as a potential conflict of interest.
